# Leprosy in wild chimpanzees

**DOI:** 10.1038/s41586-021-03968-4

**Published:** 2021-10-13

**Authors:** Kimberley J. Hockings, Benjamin Mubemba, Charlotte Avanzi, Kamilla Pleh, Ariane Düx, Elena Bersacola, Joana Bessa, Marina Ramon, Sonja Metzger, Livia V. Patrono, Jenny E. Jaffe, Andrej Benjak, Camille Bonneaud, Philippe Busso, Emmanuel Couacy-Hymann, Moussa Gado, Sebastien Gagneux, Roch C. Johnson, Mamoudou Kodio, Joshua Lynton-Jenkins, Irina Morozova, Kerstin Mätz-Rensing, Aissa Regalla, Abílio R. Said, Verena J. Schuenemann, Samba O. Sow, John S. Spencer, Markus Ulrich, Hyacinthe Zoubi, Stewart T. Cole, Roman M. Wittig, Sebastien Calvignac-Spencer, Fabian H. Leendertz

**Affiliations:** 1 Centre for Ecology and Conservation, University of Exeter, Penryn, UK; 2 Centre for Research in Anthropology (CRIA – NOVA FCSH), Lisbon, Portugal; 3 Project Group Epidemiology of Highly Pathogenic Microorganisms, Robert Koch Institute, Berlin, Germany; 4 Department of Wildlife Sciences, School of Natural Resources, Copperbelt University, Kitwe, Zambia; 5 Global Health Institute, Ecole Polytechnique Fédérale de Lausanne, Lausanne, Switzerland; 6 Department of Microbiology, Immunology and Pathology, Colorado State University, Fort Collins, CO USA; 7 Swiss Tropical and Public Health Institute, Basel, Switzerland; 8 University of Basel, Basel, Switzerland; 9 Taï Chimpanzee Project, Centre Suisse de Recherches Scientifiques, Abidjan, Côte d’Ivoire; 10 Department of Zoology, University of Oxford, Oxford, UK; 11 Department for BioMedical Research, University of Bern, Bern, Switzerland; 12 Laboratoire National d’Appui au Développement Agricole/Laboratoire Central de Pathologie Animale, Bingerville, Côte d’Ivoire; 13 Programme National de Lutte Contre la Lèpre, Ministry of Public Health, Niamey, Niger; 14 Centre Interfacultaire de Formation et de Recherche en Environnement pour le Développement Durable, University of Abomey-Calavi, Jericho, Cotonou, Benin; 15 Fondation Raoul Follereau, Paris, France; 16 Centre National d’Appui à la Lutte Contre la Maladie, Bamako, Mali; 17 Institute of Evolutionary Medicine, University of Zurich, Zurich, Switzerland; 18 Pathology Unit, German Primate Center, Leibniz‐Institute for Primate Research, Göttingen, Germany; 19 Instituto da Biodiversidade e das Áreas Protegidas, Dr. Alfredo Simão da Silva (IBAP), Bissau, Guinea-Bissau; 20 Programme National d’Elimination de la Lèpre, Dakar, Senegal; 21 Institut Pasteur, Paris, France; 22 Max Planck Institute for Evolutionary Anthropology, Leipzig, Germany; 23 Helmholtz Institute for One Health, Greifswald, Germany

**Keywords:** Anthropology, Pathogens, Bacterial infection

## Abstract

Humans are considered as the main host for *Mycobacterium leprae*^1^, the aetiological agent of leprosy, but spillover has occurred to other mammals that are now maintenance hosts, such as nine-banded armadillos and red squirrels^2,3^. Although naturally acquired leprosy has also been described in captive nonhuman primates^4–7^, the exact origins of infection remain unclear. Here we describe leprosy-like lesions in two wild populations of western chimpanzees (*Pan troglodytes verus*) in Cantanhez National Park, Guinea-Bissau and Taï National Park, Côte d’Ivoire, West Africa. Longitudinal monitoring of both populations revealed the progression of disease symptoms compatible with advanced leprosy. Screening of faecal and necropsy samples confirmed the presence of *M. leprae* as the causative agent at each site and phylogenomic comparisons with other strains from humans and other animals show that the chimpanzee strains belong to different and rare genotypes (4N/O and 2F). These findings suggest that *M. leprae* may be circulating in more wild animals than suspected, either as a result of exposure to humans or other unknown environmental sources.

## Abstract (web summary)

Monitoring of western chimpanzee populations in Guinea-Bissau and Côte d’Ivoire reveals the presence of rare and different genotypes of *Mycobacterium leprae*, suggesting greater circulation in wild animals than previously thought.

## Main

Leprosy is a neglected tropical disease caused by the bacterial pathogens *M*. *leprae* and the more recently discovered *Mycobacterium* *lepromatosis*^8,9^. In humans, the disease presents as a continuum of clinical manifestations with skin and nerve lesions of increasing severity, from the mildest tuberculoid form (or paucibacillary) to the most severe lepromatous type (or multibacillary)^10^. Symptoms develop after a long incubation period ranging from several months to 30 years, averaging 5 years in humans. As a result of sensory loss, leprosy can lead to permanent damage and severe deformity^11^. Although leprosy prevalence has markedly decreased over recent decades, approximately 210,000 new human cases are still reported every year, of which 2.3% are located in West Africa^12^. Transmission is thought to occur primarily between individuals with prolonged and close contact via aerosolized nasal secretions and entry through nasal or respiratory mucosae, but the exact mechanism remains unclear^13,14^. The role of other routes, such as skin-to-skin contact, is unknown.

Leprosy-causing bacteria were once thought to be obligate human pathogens^1^. However, they can circulate in other animal hosts in the wild, such as nine-banded armadillos (*Dasypus* *novemcinctus*) in the Americas and red squirrels (*Sciurus* *vulgaris*) in the UK^2,3^. Although initial infection was most probably incidental and of human origin, secondary animal hosts can subsequently represent a source of infection to humans^15–18^. In captivity, nonhuman primates, such as chimpanzees (*Pan* *troglodytes*)^4^, sooty mangabeys (*Cercocebus* *atys*)^5,6^ and cynomolgus macaques (*Macaca* *fascicularis*)^7^, have been known to develop leprosy without any obvious infectious source. However, due to their captive status, it is unclear how they acquired *M*. *leprae* and whether these species can also contract leprosy in the wild.

**Extended Data Fig. 1:**
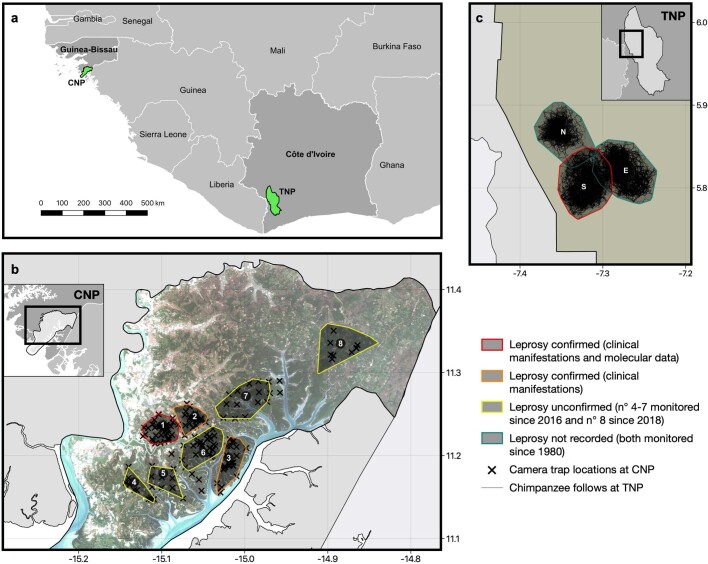
Maps of the chimpanzee study sites and chimpanzee communities. **a**, Map of the CNP, Guinea-Bissau and the TNP, Côte d’Ivoire, West Africa. **b**, Location of the chimpanzee communities at CNP that were monitored between 2015 and 2019 (1, Caiquene-Cadique; 2, Lautchande; 3, Cambeque; 4, Cabante; 5, Canamine; 6, Madina; 7, Amindara; 8, Guiledje). Estimated home ranges of chimpanzee communities at CNP are shown by 100% minimum convex polygons of direct chimpanzee observations and indirect chimpanzee traces and nests during the study period. Red outline represents chimpanzee communities with at least one individual with clinical manifestations of leprosy, confirmed using molecular analysis; orange outline represents chimpanzee communities with at least one individual with clinical manifestations of leprosy; yellow colour represents monitored communities where clinical manifestations of leprosy have not been observed nor confirmed through molecular analysis. **c**, Location of the three habituated chimpanzee communities monitored at TNP (N, north; S, south; E, east). Estimated home ranges of chimpanzee communities at TNP are shown by 100% minimum convex polygons of direct chimpanzee follows from December 2013 to October 2016. Red outline represents the community with individuals with clinical manifestations of leprosy, confirmed using molecular analysis and serological tests; blue colour represents communities where leprosy has not been recorded.

Here, we report leprosy infections and their disease course in two wild populations of western chimpanzees (*P*. *troglodytes verus*) in Cantanhez National Park (CNP), Guinea-Bissau, and in Taï National Park (TNP), Côte d’Ivoire, using a combination of camera trap and veterinary monitoring (Extended Data Fig. 1a and Supplementary Notes 1 and 2). From analyses of faecal samples and postmortem tissues, we identified *M*. *leprae* as the causative agent of the lesions observed and determined the phylogenetic placement of the respective strains based on their complete genome sequences.

**Fig. 1:**
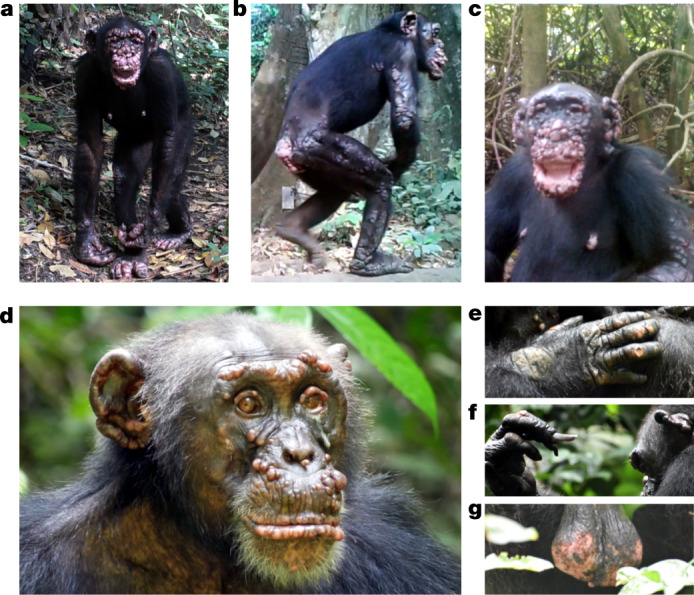
Clinical manifestations of leprosy in three chimpanzees at CNP, Guinea-Bissau and TNP, Côte d’Ivoire. **a–c**, Clinical signs of leprosy in two adult female chimpanzees in CNP (images extracted from camera traps). **a**, Rita has large hypopigmented nodules covering the entire body; disfigurement of the face, ears, hands and feet (ulcerated lesions and swelling). **b**, Rita has extensive plaques covering all limbs, with hair loss. **c**, Brinkos has large hypopigmented nodules covering the entire face, with extreme disfigurement of the face and ears, and ulcerated plaques on the arms and the nipples. **d**–**g**, Clinical signs of leprosy in an adult male chimpanzee, Woodstock, at TNP. **d**, Multiple hypopigmented nodules on the ears, brow ridges, eyelid margins, nostrils, lips and the area between the upper lip and the nose. **e**, Hypopigmentation and swelling of the hands with ulcerations and hair loss on the dorsal side of the joints. **f**, Claw hand with nail loss and abnormal overgrowth of fingernails. **g**, Scrotal reddening and ulceration with fresh blood.

**Extended Data Fig. 2:**
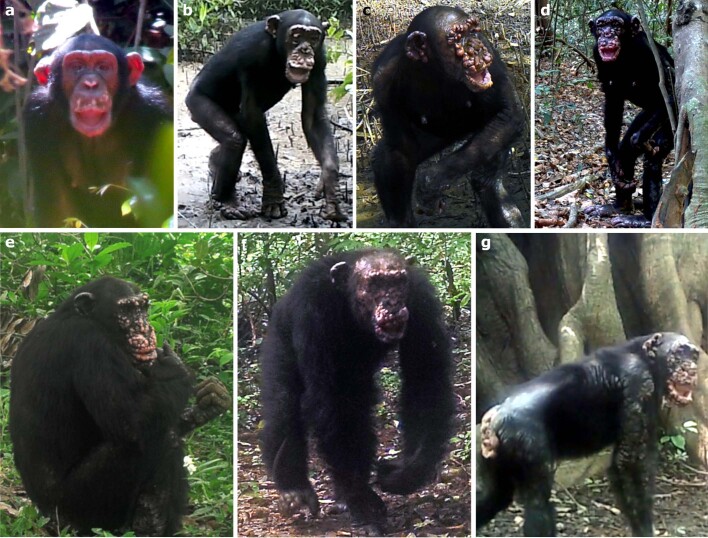
Disease progression of leprosy in chimpanzees at CNP. Adult female chimpanzee Rita over the course of 5 years (**a**–**d**) and disease manifestations in three additional adult chimpanzees (**e**–**g**). **a**, 2013/05 – Hypopigmentation of skin around the mouth and nose, small nodule on the lower lip and left ear (opportunistically recorded with a video camera before the start of longitudinal health monitoring with camera traps). **b**, 2015/12 – Large nodules between the upper lip and nose, with multiple small nodules on the eyelids, cheek, ears margins, lower lip and brow ridge. Small dry patches with hair loss on the wrists, knees and elbows. **c**, 2017/12 – Nodules increase in number, with apparent swelling and reddening, facial disfigurement and claw hand. Plaques appear on the wrist, knee and elbow joints, with an increase in hair thinning. **d**, 2018/05 – Face and ears completely covered by large nodules, with facial disfigurement and generalized hair loss on limbs and lower back. Nodule formation and swelling of fingers and toes, with disfigurement of hands and feet, and more severe claw hand. Some plaques on the body are ulcerated, and the individual has clear weight loss. **e**, Jimi (Lautchande in 2018/06) – First observation of lesions in 2015. The head is completely covered with multiple nodules of reddish colour, some of which are ulcerated. Ear margins are thickened. Hands and feet present nodules and plaques, and the scrotum is affected (not visible on picture). **f**, Baaba (Cambeque in 2017/08) – First observation of lesions in 2017. Multiple hypopigmented nodules on the brow ridge, cheek and upper and lower lips. Ears have thickened margins and nodules. There is hair thinning, with multiple small plaques present on the upper and lower limbs, back, abdomen and shoulders. **g**, Brinkos (Caiquene-Cadique in 2018/10) – First observation of lesions in 2015. Facial disfigurement, with the ulceration of nodules and a hanging lower lip. Hands and feet are ulcerated, and fingers are swollen. There are nodules on the nipples, and plaques covering the lower back, shoulders and arm are ulcerated, with hair loss.

Chimpanzees at CNP are not habituated to human observers, precluding systematic behavioural observations. Longitudinal studies necessitate the use of camera traps, which we operated between 2015 and 2019. Of 624,194 data files (videos and photographs) obtained across 211 locations at CNP (Extended Data Fig. 1b, Extended Data Table 1 and Supplementary Table 1), 31,044 (5.0%) contained chimpanzees. The number of independent events (images separated by at least 60 min) totalled 4,336, and of these, 241 (5.6%) contained chimpanzees with severe leprosy-like lesions, including four clearly identifiable individuals (two adult females and two adult males) across three communities (Extended Data Fig. 2 and Supplementary Note 2). As with humans, paucibacillary cases in chimpanzees may be present but easily go undetected. Such minor manifestations of leprosy are not reported. All symptomatic chimpanzees showed hair loss and facial skin hypopigmentation, as well as plaques and nodules that covered different areas of their body (limbs, trunk and genitals), facial disfigurement and ulcerated and deformed hands (claw hand) and feet (Fig. 1a–c), consistent with a multibacillary form of the disease. Longitudinal observations showed progression of symptoms across time with certain manifestations similar to those described in humans (such as progressive deformation of the hands) (Extended Data Fig. 2 and Supplementary Videos 1–3). To confirm infection with *M*. *leprae*, we collected faecal samples and tested them with two nested polymerase chain reaction (PCR) assays targeting the *M*. *leprae-*specific repetitive element (RLEP) and 18 kDa antigen gene. One out of 208 DNA extracts from CNP was positive in both assays and a second was positive only in the more sensitive RLEP-PCR^19^ (Extended Data Table 2, Supplementary Table 2 and Supplementary Note 3). Microsatellite analyses of the two positive samples confirmed that they originated from two distinct female individuals (Supplementary Note 4 and Supplementary Tables 3 and 4). Our results suggest that *M*. *leprae* is the most likely cause of a leprosy-like syndrome in chimpanzees from CNP.

**Extended Data Fig. 3:**
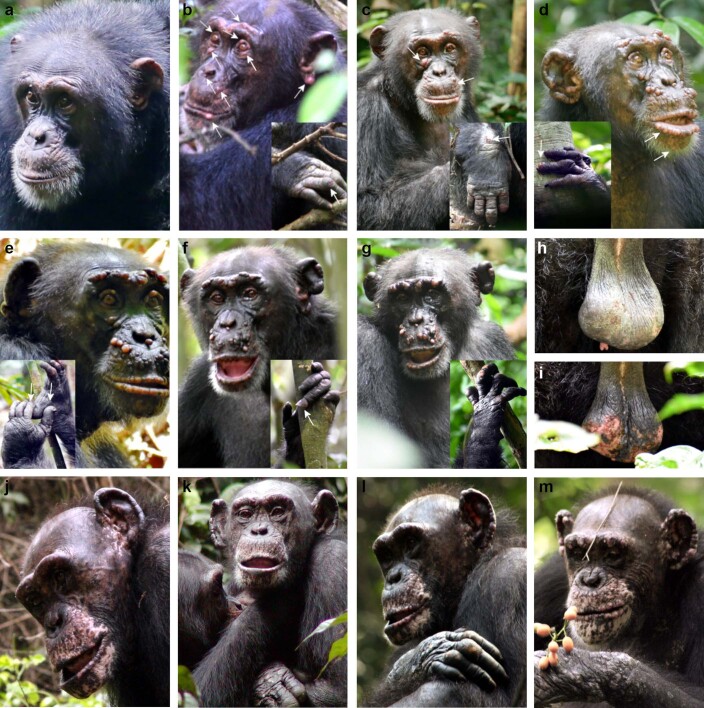
Disease progression of leprosy in an adult male chimpanzee at TNP (Woodstock) over the course of 2 years (2018–2020) (**a**–**i**) and an adult female chimpanzee at TNP (Zora) over the course of 2008–2009 (**j**–**m**). **a**, 2017/01 – Woodstock before the appearance of clinical signs. **b**, 2018/06 – First hypopigmented nodules appear on the face (arrows), with swelling and hypopigmentation on both hands, and ulceration on the right hand. **c**, 2018/10 – Existing nodules increase in size and new smaller ones appear (arrows). Development of mucopurulent discharge from the left eye, and lower eyelid is turned outward. Hair loss and ulceration on dorsal part of right wrist and hand. **d**, 2019/04 – Most existing nodules increase in size and become pedunculated, and the nodule under the eye shrinks, and several new nodules appear (see arrows). Suspected start of nasal involvement, and right ear starts to become disfigured. Both hands are slightly swollen and hypopigmented, with the loss of nail plate on the fourth finger of the left hand, and the third and fifth fingers show early stage of abnormal nail overgrowth. **e**, 2019/10 – Facial lesions increase in size, and some become darkly pigmented. New lesions appear on the brow ridge, with nodules above the lips and between the lips, and the nose becomes pedunculated. The loss of nail plate, and nail bed becomes exposed on the first and second fingers of the left hand. **f**, 2020/04 – In general, facial nodules seem smaller than before, and the nodule under the left eye disappears. On the left hand, the nail of the fourth finger shows an advanced stage of abnormal nail overgrowth, and the third and fifth fingernails show early stage of abnormal nail overgrowth. **g**, 2020/07 – Facial nodules seem larger with many hypopigmented, and both ears are swollen and disfigured. Nasal involvement becomes apparent. Both hands are swollen and hypopigmented. Skin ulcerations present on the right hand, with possible claw hand on the left hand. **h**, 2019/04 – Slight hypopigmentation of scrotum. **i**, 2020/07 – Reddening and ulceration of scrotum; fresh blood observed. **j**, 2007/12 – Zora before the appearance of clinical signs of leprosy. **k**, 2008/01 – Appearance of nodules on the right ear and both eyebrow ridges. **l**, 2008/12 – Appearance of nodules on the left ear, and ulceration of the skin at the second, third and fourth proximal interphalangeal joint level of the right hand. **m**, 2009/04 – Nodular lesions on both ears and brow ridge seem aggravated, with nodular lesions on the lips, and above the mouth (four months before death).

At TNP, chimpanzees are habituated to the presence of researchers and have been followed daily since 1979. In addition, necropsy samples have been collected from all dead individuals recovered since 2000. In June 2018, researchers first noticed leprosy-like lesions on Woodstock, an adult male chimpanzee from one of the three habituated communities (south) (Extended Data Fig. 1c). The initial small nodules on the ears, lips and under the eye became more prominent and were followed by nodules on the eyebrows, eyelids, nostrils, ears, lips and face. The skin on facial nodules, hands, feet and testicles became hypopigmented and the loss and abnormal growth of nails was observed (Fig 1d–g, Extended Data Fig. 3 and Supplementary Videos 4 and 5). *Mycobacterium leprae* DNA was detected in all samples from June 2018 (Extended Data Table 2, Supplementary Table 2 and Supplementary Note 2). Here, continuous noninvasive detection of *M. leprae* was associated with the onset and evolution of a leprosy-like disease.

**Extended Data Fig. 4:**
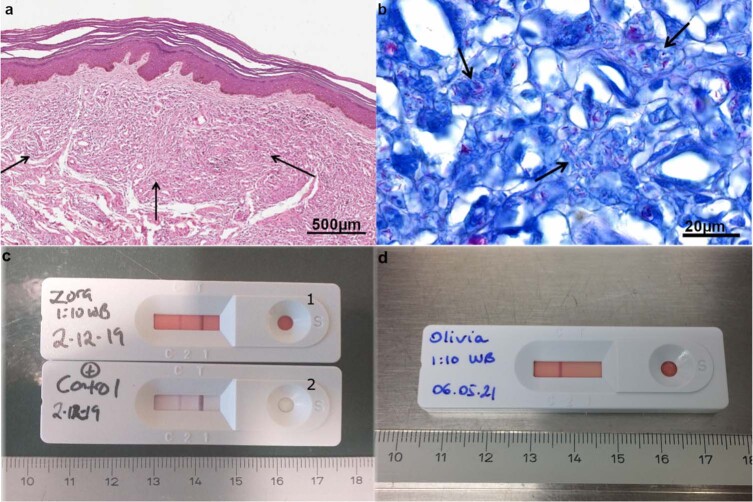
Confirmation of leprosy infection in Zora through histopathology of skin sample and lateral flow test. **a**, Lepromatous leprosy, skin with diffuse histiocytic infiltrate in the dermis. The haematoxylin and eosin stain was conducted once; scale bar, 500 μm. **b**, Lepromatous leprosy, skin, acid-fast bacilli in histiocytes. The inflammatory infiltrate consists predominantly of histiocytes admixed with fewer lymphocytes. Histiocytes show foamy or vacuolated cytoplasm and containing bacteria surrounded by a clear zone. Fite-Faraco stain; scale bar, 20 μm. Fite-Faraco stain was conducted once and was controlled by a positive control slide containing mycobacteria. **c**, whole blood from Zora (1) and the positive control (2). **d**, whole blood from a chimpanzee at TNP (Olivia) not infected with *M. leprae*, used as negative control. C, control lane; T, test lane.

Retrospective PCR screening of all chimpanzee spleen samples (*n* = 38 individuals) from the TNP necropsy collection led to the identification of *M. leprae* DNA in two further individuals. An adult female from the same community named Zora, who had been killed by a leopard in 2009, tested positive in both PCR assays. The presence of *M. leprae* DNA was confirmed by PCR in various other organs (Extended Data Table 2). Retrospective analyses of photographs taken in the years before her death showed progressive skin hypopigmentation and nodule development since 2007 (Extended Data Fig. 3). Formalin-fixed skin samples (hands and feet) were prepared for histopathological examination using haematoxylin and eosin as well as Fite-Faraco stains. The skin presented typical signs of lepromatous leprosy characterized by a diffuse cutaneous cell infiltration in the dermis and the subcutis clearly separated from the basal layer of the epidermis (Extended Data Fig. 4a). We detected moderate numbers of acid-fast bacilli (single or in clumps) within histiocytes, indicative of *M. leprae* (Extended Data Fig. 4b). As antibodies against the *M. leprae*-specific antigen phenolic glycolipid-I (PGL-I) are a hallmark of *M. leprae* infection in humans^20^, we also performed a PGL-I lateral flow rapid test^21^ on a blood sample from this individual, which showed strong seropositivity (Extended Data Fig. 4c). Faecal samples collected in the years before Zora’s death contained *M. leprae* DNA from 2002 onwards, implying at least 7 years of infection (Extended Data Table 2). In this case, disease manifestations, histopathological findings, serological and molecular data, as well as the overall course of the disease, all unambiguously point towards *M. leprae*-induced leprosy.

To ascertain whether other individuals in the south community of TNP were infected at the time of Zora’s death in 2009, cross-sectional screening of contact animals (*n* = 32) was performed by testing all available faecal samples (*n* = 176) collected in 2009 (Supplementary Table 2). Three other chimpanzees were PCR-positive in single samples, including Woodstock. Clinical symptoms of leprosy have not been observed in other individuals, despite daily monitoring of south community members for 20 years and of neighbouring communities for 40 years^22,23^. Considering that, over this period, 467 individuals have been observed, it seems that leprosy is a rare disease with low transmission levels in these chimpanzee communities.

**Extended Data Fig. 5:**
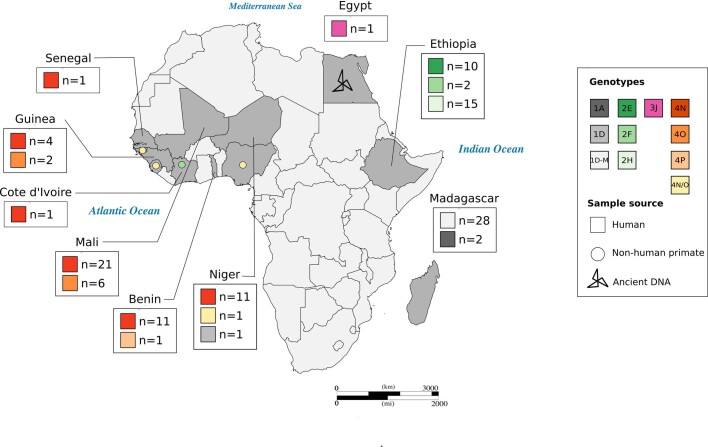
Geographical distribution of *M. leprae* genotypes in Africa based on genome data. The genotype 2F has never been reported in West Africa and is the least identified in Ethiopia. The genotype 4N/O was only reported in one human sample from West Africa. Data included only *M. leprae* genomes (Supplementary Note 5 and Supplementary Table 7). The map was downloaded from https://www.amcharts.com/svg-maps/ under a free licence and modified for the current figure in Inkscape, an open source digital illustration software package (https://inkscape.org).

To characterize the *M*. *leprae* strains causing leprosy in wild chimpanzees and to perform phylogenomic comparisons, we selected DNA extracts that were positive in both the RLEP and the less-sensitive 18-kDa PCR, which indicates relatively high levels of *M*. *leprae* DNA. For TNP, we selected individuals that were positive in multiple samples. Following targeted enrichment using hybridization capture, samples were subjected to Illumina sequencing. Sufficient *M*. *leprae* genome coverage was obtained for sample GB-CC064 (Guinea-Bissau) and for Zora (Côte d’Ivoire) with mean depth of 39.3× and 25.8×, respectively (Extended Data Table 2 and Supplementary Table 5). We generated 21 *M*. *leprae* genomes from human biopsies from five West African countries (Niger, Mali, Benin, Côte d’Ivoire and Senegal) and depth of coverage ranged from 4.7× to 170×. We assembled a dataset that included the genomes generated in this study and all previously available *M*. *leprae* genomes. Of the total 286 genomes, 64 originated from six West African countries (Extended Data Fig. 5 and Supplementary Note 5).

**Fig. 2:**
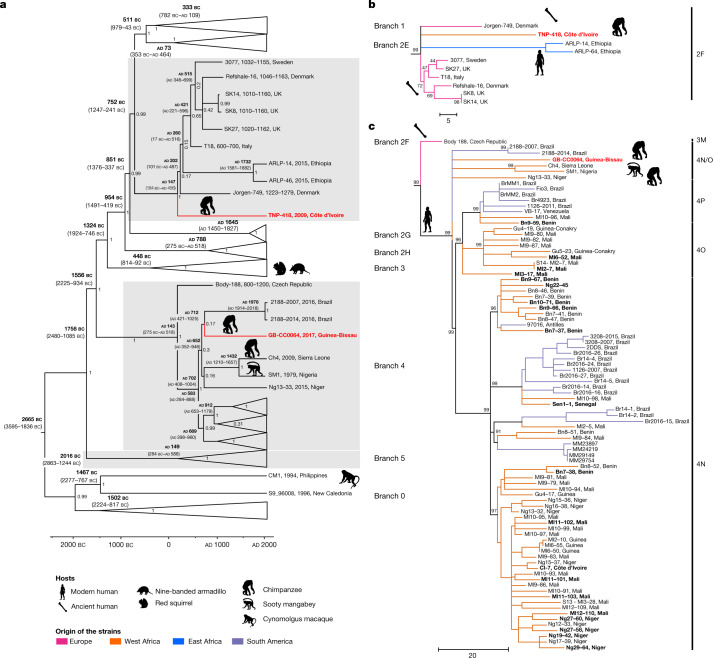
Phylogeny of *M. leprae* strains from human and animal hosts. **a**, Bayesian dated phylogenetic tree of 278 *M. leprae* genomes including the two new chimpanzee strains (in bold red). Hypermutated samples with mutations in the *n*th gene were excluded from the analysis. The tree is drawn to scale, with branch lengths representing years of age. Median estimates of node ages are shown in black above branches; 95% HPD intervals are shown in grey. Some *M. leprae* branches are collapsed to increase readability. **b**, Maximum parsimony tree of branch 2F. **c**, Maximum parsimony tree of the branch 4. The tree was initially constructed using 286 genomes (Supplementary Table 6), including 2 new chimpanzee strains (in bold red) and 21 new genomes from West Africa (in bold), 500 bootstrap replicates and *M*. *lepromatosis* as outgroup. Sites with missing data were partially deleted (80% genome coverage cutoff), resulting in 4,470 variable sites used for the tree calculation. Subtrees corresponding to branches were retrieved in MEGA7^65^. Corresponding genotypes are indicated on the side of each subtree. Samples are binned according to geographical origin as given in the legend. Scale bars (**b**, **c**), number of nucleotide substitutions. Animal silhouettes are available under Public Domain licence at PhyloPic (http://PhyloPic.org/).

**Extended Data Fig. 6:**
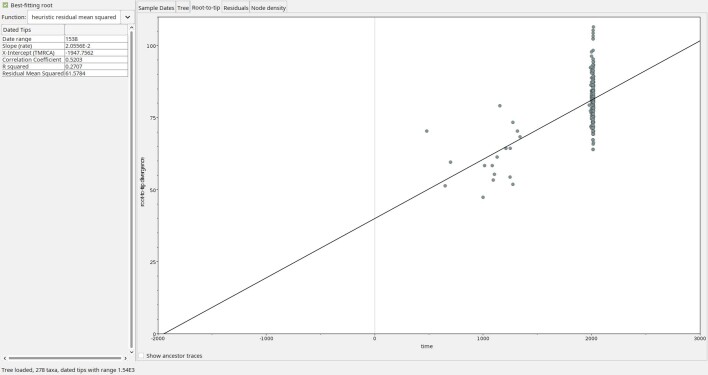
Best-fitting root analysis using TempEst.

**Extended Data Fig. 7:**
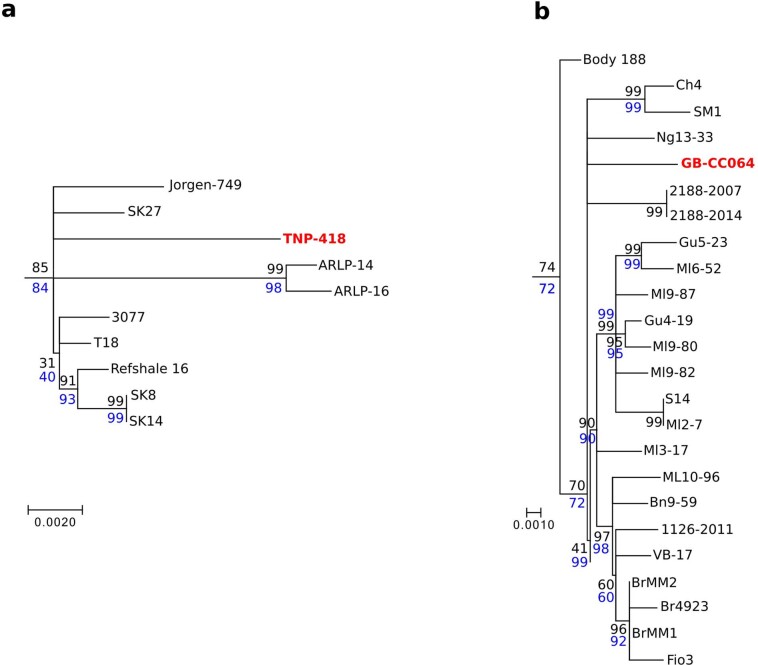
Maximum Likelihood tree to confirm the topological placement of GB-CC064 and TNP-418. 286 genomes (Supplementary Table 6) were used, including the two new chimpanzee strains (in bold red), 500 bootstrap replicates (value in black with the Tamura Nei model and in blue for the general time model) and *M. lepromatosis* as outgroup. Sites with missing data were partially deleted (80% genome coverage cutoff). **a**, Maximum Likelihood tree of the branch 2F. **b**, Maximum Likelihood tree of the branch 4.

Bayesian and maximum-parsimony analyses (Extended Data Figs. 6 and 7) place the strain from Guinea-Bissau (GB-CC064) on branch 4, where it clusters outside the standard genotypes 4N, 4O and 4P, but within the so-called 4N/O genotype^24,25^ (Fig. 2a, c). This 4N/O genotype is rare and only comprises five *M. leprae* strains; one strain (Ng13-33) from a patient in Niger, two strains (2188-2007 and 2188-2014) obtained from a single patient in Brazil (of 34 strains in Brazil)^26^ and two strains from two captive nonhuman primates originating from West Africa (Ch4 and SM1)^25^. The branching order of these five strains and GB-CC064 was unresolved in our analyses, with a basal polytomy suggestive of star-like diversification within this genotype, and within the group comprising all genotype 4 strains (4N/O, 4N, 4P and 4O). Divergence from the most recent common ancestor for this group is estimated to have occurred in the sixth century ad (mean divergence time, 1,437 years ago, 95% highest posterior density (HPD) 1,132–1,736 years ago). The strain that infected Zora in Côte d’Ivoire, designated TNP-418, belongs to branch 2F, within which, the branching order was also mostly unresolved (Fig. 2a, b). The branch is currently composed of human strains from medieval Europe (*n* = 7) and modern Ethiopia (*n* = 2), and this genotype has thus far never been reported to our knowledge in West Africa. Bayesian analysis estimated a divergence time during the second century ad (mean of 1,873 years ago (95% HPD 1,564–2,204 years ago)), similar to previous predictions^27^.

Samples from Woodstock did not yield enough Illumina reads to reconstruct full genomes for phylogenomic analysis. However, single-nucleotide polymorphisms (SNPs) recovered from the few available Illumina reads and Sanger sequences derived from PCR products allowed us to assign this second *M. leprae* strain from Côte d’Ivoire to the same genotype as TNP-418 (Supplementary Note 5). Overall, phylogenomic analyses show that *M. leprae* strains in chimpanzee populations at CNP and TNP are not closely related.

The finding of *M. leprae*-induced leprosy in wild chimpanzee populations raises the question of the origin(s) of these infections. *Mycobacterium leprae* is considered a human-adapted pathogen and previous cases of leprosy affecting wildlife were compatible with anthroponosis. Therefore, the prime hypothesis would be human-to-chimpanzee transmission. Potential routes of transmission include direct (such as skin-to-skin) contact and inhalation of respiratory droplets and/or fomites, with the assumption that, in all cases, prolonged and/or repeated exposure is required for transmission^11^. Chimpanzees at CNP are not habituated to humans and are not approached at distances that would allow for transmission via respiratory droplets. Although these chimpanzees inhabit an agroforest landscape and share access to natural and cultivated resources with humans^28^, present-day human–chimpanzee direct contact is uncommon. The exact nature of historic human–chimpanzee interactions at CNP remains, however, unknown. For example, robust data on whether chimpanzees were kept as ‘pets’ or were hunted for meat are lacking. Long-term human–chimpanzee coexistence in this shared landscape makes humans the most probable source of chimpanzee infection. However, multiple individuals from several chimpanzee communities across CNP show symptomatic leprosy demonstrating that *M. leprae* is now probably transmitted between individuals within this population.

At TNP, the south chimpanzee community is distant from human settlements and agriculture. Human-to-animal transmission of pathogens has been shown at TNP^29,30^ but involved respiratory pathogens (pneumoviruses and human coronavirus OC43) that transmit easily and do not require prolonged exposure. In addition, *M. leprae* is thought to be transmitted from symptomatic humans^31^ and no cases of leprosy have been reported among researchers or local research assistants. Although a human source is impossible to rule out, low human contact coupled with the rarity of the *M. leprae* genotype detected in TNP chimpanzees among human populations in West Africa suggests that recent human-to-chimpanzee transmission is unlikely. This is supported by the absence of drug-resistant mutations (Supplementary Note 6). The relatively old age of the lineage leading to the chimpanzee strain at TNP nevertheless raises the possibility of an ancient human-to-chimpanzee transmission. However, the human population density 1,500–2,000 years ago was probably even lower than it is currently, making this unlikely. If such an ancient transmission had occurred and the bacterium had persisted for a long time in chimpanzees, it should have spread more broadly as observed in *M. leprae*-infected squirrels and armadillos^3,16,17^. Therefore, an ancient human-to-chimpanzee transmission is not the most plausible mechanism to explain the presence of *M. leprae* in chimpanzees at TNP.

These findings may be better explained by the presence of a nonhuman leprosy reservoir. As chimpanzees hunt frequently, transmission may originate from their mammalian prey^32^. Nonhuman primates are the most hunted prey at TNP^33^ and are hunted at CNP (Supplementary Note 3). Chimpanzees also consume other mammalian prey such as ungulates. Notably, this scenario assumes that the animal host range of *M. leprae* is even broader than is currently known. Perhaps more intriguingly, an environmental source may be at the origin of chimpanzee infections. Other mycobacteria can survive in water, including *M. ulcerans* and other non-tuberculous mycobacteria^34,35^, and molecular investigations have reported that *M. leprae* can survive in soil^36^. Experimental data also show that *M. leprae* multiplies in amoebae^37^, arthropods^38^ and ticks^39^, which could contribute to the persistence of the bacteria in the environment. Testing these hypotheses will require thorough investigation of the distribution of *M. leprae* in wildlife and the environment and so shed light on the overall transmission pathways of the pathogen.

## Methods

### Study sites

Observational study and sample collections were performed at CNP in southern Guinea-Bissau and TNP in western Côte d’Ivoire (Extended Data Fig. 1a). CNP (1,067 km^2^) comprises the Cubucaré peninsula in the sector of Bedanda, with the northeast of the park bordering the Republic of Guinea. The landscape at CNP consists of a mosaic of mainly mangroves, subhumid forest patches, savannah grassland and woodland, remnant forest strips dominated by palm groves as well as agriculture^40^. There are approximately 200 villages and settlements within the borders of the park, with an estimated human population of 24,000 individuals who comprise several ethnic groups^41^. Chimpanzees are not hunted for consumption within CNP due to local cultural beliefs and taboos^42^ but are sometimes killed in retaliation for foraging on crops^43,44^. There is a minimum of 12 chimpanzee communities at CNP^41^, all unhabituated to researchers, with approximately 35–60 individuals per community^45,46^. Numerous other wildlife taxa inhabit CNP, including six other nonhuman primate species^41,47^.

The TNP (5,082 km^2^) consists of an evergreen lowland rainforest and is the largest remaining primary forest fragment in West Africa. It is home to a wide range of mammals that include 11 different nonhuman primate species^48,49^. There are no settlements or agricultural areas inside the National Park. As of March 2021, the three habituated communities, north, south and east, comprised 22, 37 and 32 individuals, respectively, although community sizes have varied over time. Systematic health monitoring of these communities has been ongoing since 2000^23^.

### Longitudinal observations and health monitoring

At CNP, camera traps (Bushnell Trophy Cam models 119774, 119877 and 119875) were deployed at 211 locations, including across different habitat types (forest, mangrove-forest edge and orchards) within the home range of 8 of the 12 putative chimpanzee communities (Supplementary Table 1). Camera traps were set up over six data collection periods from 2015 to 2019 (Extended Data Table 1). Targeted camera traps were deployed to record and monitor chimpanzee behaviour and disease occurrence. To maximize the chances of recording specific behaviours and to identify leprosy-like symptoms in individuals, targeted camera traps were set up in locations that chimpanzees were known to use most often, sometimes in clusters, precluding uniform survey designs. Targeted camera traps were set up in video mode and were active 24 h per day. When triggered, targeted cameras recorded 10 to 60 s of video with a minimum interval of 0.6 s or 2 s, depending on the camera trap model. Furthermore, systematically placed camera traps were used to obtain measures of wildlife occurrence and habitat use across the heterogeneous landscape^41^. Systematic camera traps were deployed across central CNP, at a minimum distance of 1 km between sampling points, as well as within the home range of one chimpanzee community (Caiquene-Cadique) and were spaced at least 500 m from one another. The camera traps pointed towards animal paths (often chimpanzee paths), small human paths also used by wildlife and other areas presenting signs of animal activity. Systematic camera traps were set up to record three consecutive photographs when triggered. The GPS coordinates, habitat type, date, time and site description were recorded when setting up individual camera traps (targeted and systematic). Opportunistic observations of chimpanzees at CNP were made in 2013, during which chimpanzees were photographed and/or filmed using digital cameras.

Chimpanzees at TNP are fully habituated to human observers and all individuals in the habituated communities are individually identified. Behavioural and health monitoring of chimpanzees at TNP involves daily observation of habituated individuals by an interdisciplinary team comprising primatologists and veterinarians; investigations of wildlife mortality causes through necropsies on all animal carcasses found in the research area; and the collection of noninvasive samples such as faecal samples, laboratory investigations and the communication of the results to the park management for corrective and preventive measures^22^. Abnormalities in behaviour or clinical signs of disease are immediately reported and followed by detailed observation by the on-site veterinarian. To reduce the risk of transmission of human diseases to the chimpanzees, stringent hygiene measures have been put in place, including an initial 5-day quarantine for observers, keeping a distance of at least 7 m and obligatory wearing of masks, with only healthy observers allowed to work in the forest^50,51^.

### Faecal and necropsy sample collection

At CNP, chimpanzee faecal samples were collected between July 2017 and December 2018. The date and putative chimpanzee community were recorded for each faecal sample. As defecation was rarely observed and to prevent the collection of redundant samples from the same individual, we avoided multiple samples found under the same chimpanzee nest and paid special attention if multiple samples were found in proximity on trails^45,52,53^. All samples were collected with the aid of a wooden spatula and stored at ambient temperature in 15-ml tubes containing NAP buffer^54^. All samples were sent to the Robert Koch Institute for laboratory analysis. Even though chimpanzee faeces are easily distinguishable from those of other species and were found in areas where chimpanzees had recently been present with associated signs such as feeding remains or knuckle prints, we genetically confirmed the presence of chimpanzee DNA in faecal samples that tested positive in either of the *M. leprae* PCRs or the mammal PCR for diet analysis (Supplementary Note 3).

At TNP, the long-term health monitoring programme includes continuous collection of faecal and urine samples from known adult chimpanzees. Faeces are collected right after defecation, transferred to 2-ml cryotubes with the aid of a plastic spatula and frozen in liquid nitrogen the same day. A full necropsy is systematically performed on chimpanzees found dead by the on-site veterinarian. Necropsies follow a standardized biosafety protocol due to the occurrence of anthrax, Ebola and monkeypox in the area. This includes the use of full personal protective equipment and rigorous disinfection measures. Tissue samples of several internal organs are taken if the state of carcass decomposition allows. After collection, all samples are first stored in liquid nitrogen and subsequently shipped on dry ice to the Robert Koch Institute for analyses.

### DNA extraction from faeces and necropsy samples

DNA extractions were performed at the Robert Koch Institute in a laboratory that has never been used for molecular *M. leprae* investigations. DNA was extracted from faecal and necropsy samples using the GeneMATRIX stool DNA purification kit (EURx) and the DNeasy Blood and Tissue kit (QIAGEN), respectively, following the manufacturers’ instructions. Extracted DNA was then quantified using the Qubit dsDNA HS Assay kit (Thermo Fisher Scientific) and subsequently stored at −20 °C until further use.

### Genetic identification of samples from infected chimpanzees at CNP

To determine whether faecal samples positive for *M. leprae* belonged to one or two individuals at CNP, we amplified chimpanzee DNA at 11 microsatellite loci and one sexing marker^55^. Owing to the small quantity of starting DNA, not all loci were amplified and in some cases the amplification quality was low, affecting our ability to confidently interpret allele peak profiles (for example, sample GB-CC064 failed to amplify for 5 out of the 11 loci) (Supplementary Note 4).

### Molecular screening of *M. leprae* in faecal and necropsy samples

*Mycobacterium leprae* DNA was searched for using two nested PCR systems targeting the distinct but conserved repetitive element RLEP and the 18-kDa antigen gene as previously described (Extended Data Table 3). As 37 copies of RLEP are present in the *M. leprae* genome, this assay is considered to be more sensitive than 18 kDa, for which there is only a single copy. To prevent contamination at the laboratory at the Robert Koch Institute and to enable us to identify whether it occurs, we followed these procedures: (1) separate rooms were used for preparation of PCR master mixes and the addition of DNA in the primary PCR; (2) the addition of the primary PCR product in the nested PCR in another separate room; and (3) dUTPs were used for all PCRs instead of dNTPs. For both assays, primary PCRs were performed in 20-µl reactions: up to 200 ng of DNA was amplified using 1.25 U of high-fidelity Platinum Taq polymerase (Thermo Fisher Scientific), 10× PCR buffer, 200 µM dUTPs, 4 mM MgCl_2_ and 200 nM of both forward and reverse primers. The thermal cycling conditions for the primary and nested PCRs were as follows: denaturation at 95 °C for 3 min, followed by 50 cycles of 95 °C for 30 s, 55 °C (18 kDa primers) or 58 °C (RLEP primers) for 30 s, and 72 °C for 1 min as well as an elongation step at 72 °C for 10 min. For nested PCRs, 2 µl of a 1:20 dilution of the primary PCR product was used as a template. Molecular-grade water was used as a template-free control. PCR products were visualized on a 1.5% agarose gel stained with GelRed (Biotium). Bands of the expected size were purified using the Purelink Gel extraction kit (Thermo Fisher Scientific). Both RLEP and 18-kDa nested PCR products are too short for direct Sanger sequencing. Therefore, fusion primers (primary PCR primers coupled with M13F and M13R primers) (Extended Data Table 3) were used for further amplification of the cleaned PCR products, applying the same conditions as in the primary PCR, but running only for 25 cycles. The resulting extended PCR products were then enzymatically cleaned using the ExoSAP-IT PCR Product Cleanup assay (Thermo Fisher Scientific) and Sanger sequenced using M13 primers. Resulting sequences were compared to publicly available nucleotide sequences using the Basic Local Alignment Search Tool (BLAST)^56^.

### Histopathology

To further confirm the infection, skin samples were sent to the German Primate Center in Göttingen, Germany for histopathological analyses. Samples were immersion-fixed in 10% neutral-buffered formalin, embedded in paraffin and stained with standard haematoxylin and eosin using the Varistain Gemini staining automat (Thermo Fisher Scientific). Samples were also stained with Fite-Faraco stain for the identification of acid-fast bacilli.

### Serology

A whole-blood sample from Zora collected during the necropsy in 2009 was tested for the presence of the *M. leprae*-specific anti-PGL-I antibodies using a chromatographic immunoassay developed for use with human blood following the instructions provided by the test manufacturers with a 1:10 diluted whole-blood sample. This rapid lateral flow test was produced by R. Cho using the synthetic ND-O-BSA antigen with financial support of the NIH/NIAID Leprosy Research Materials contract AI-55262 at Colorado State University. Test results were interpreted at 5 and 10 min. Human serum from a patient with multibacillary leprosy donated by J. S. Spencer, Colorado State University, was used as a positive control. Whole blood collected during the necropsy of a chimpanzee (Olivia) at TNP who died of acute respiratory disease in 2009 was used as a negative control.

### Library preparation, genome-wide capture and high-throughput sequencing for nonhuman primate samples

Selected *M. leprae-*positive faecal and necropsy samples (Supplementary Table 2) were converted into dual-indexed libraries using the NEBNext Ultra II DNA Library Prep kit (New England Biolabs)^57,58^. To reconstruct whole genomes, libraries were target-enriched for *M. leprae* DNA using in-solution hybridization capture with 80-nt RNA baits designed to cover the whole *M. leprae* genome (twofold tiling; design can be shared upon request to the corresponding author) and following the myBaits protocol as previously described^25^. Around 1.5 µg of each DNA library was captured in single or pooled reactions. Two rounds of 24-h hybridization capture were performed followed by a post-amplification step for each using the KAPA HiFi HotStart Library Amplification kit with 12 to 16 cycles to generate around 200 ng of enriched library per sample. Finally, enriched libraries were purified using the silica-based MinElute reaction cleanup kit (QIAGEN) followed by quantification with the KAPA library quantification kit (Roche). Libraries were then normalized and pooled across sequencing lanes on an Illumina NextSeq 500 for sequencing with a mid-output kit v.2 for 300 cycles (Illumina).

### Sample collection, DNA extraction, library preparation, genome-wide capture and high-throughput sequencing of human specimens

Samples (skin biopsies or DNA extracts) from patients with leprosy from five West African countries who had a positive bacillary index (Niger (*n* = 5), Mali (*n* = 8), Benin (*n* = 6), Côte d’Ivoire (*n* = 1) and Senegal (*n* = 1)) were obtained from the respective National Leprosy Control Programmes in the framework of the leprosy drug-resistance surveillance programmes or from previous investigation^59^.

DNA was extracted from skin biopsies using the total DNA extraction method as described previously^60^. DNA was quantified with a Qubit fluorometer using the Qubit dsDNA BR Assay kit (Thermo Fisher Scientific) before library preparation. DNA libraries were prepared using the KAPA Hyper Prep kit (Roche) as per the manufacturer’s recommendation using KAPA Dual-Indexed Adapter (Roche) followed by in-solution capture enrichment with 80-nt RNA baits with 2× tiling density for 48 h at 65 °C as described previously^60^. Post-capture amplification was performed with seven cycles. Enriched libraries were purified using a 1× ratio of KAPA Pure beads (Roche) followed by quantification with the KAPA library quantification kit (Roche) and quality control of the fragment with the Agilent 2200 TapeStation (Agilent Technologies). Libraries were then normalized and pooled across sequencing lanes on an Illumina NextSeq 500 for sequencing with a high output kit v.2 for 75 cycles (Illumina).

### Genomic data analysis

Raw reads were processed as described elsewhere^24^. Putative unique variants of GB-CC064 and TNP-418 strains were manually checked and visualized using the Integrative Genomics Viewer^61^.

### Genome-wide comparison and phylogenetic tree

SNPs of the two newly sequenced genomes from chimpanzees were compared to the 263 publicly available *M*. *leprae* genomes^25,60,62–64^ (Supplementary Table 6) and 21 new genomes from West African countries (Supplementary Note 5). Phylogenetic analyses were performed using a concatenated SNP alignment (Supplementary Table 7). Maximum-parsimony trees were constructed in MEGA7^65^ with the 286 genomes available (Supplementary Table 5) using 500 bootstrap replicates and *M*. *lepromatosis*^66^ as outgroup. Sites with missing data were partially deleted (80% genome coverage cutoff), resulting in 4,470 variable sites used for the tree calculation.

### Dating analysis

Dating analyses were performed using BEAST2 (v.2.5.2)^67^ as described previously^24^ with 278 genomes and an increased chain length from 50 to 100 million. In brief, concatenated SNPs for each sample were used for tip dating analysis (Supplementary Table 7). Hypermutated strains and highly mutated genes associated with drug resistance (in yellow, Supplementary Table 7) were omitted^24,60^, manual curation of the maximum parsimony and BEAST input file was conducted at the positions described in Supplementary Table 9 for GB-CC064 and TNP-418. Sites with missing data as well as constant sites were included in the analysis, as previously described^24^. Only unambiguous constant sites (loci where the reference base was called in all samples) were included.

### PCR genotyping of insufficiently covered *M*. *leprae* genomes from positive chimpanzees

The genome coverage for the strain infecting Woodstock was low. To be able to determine the genotype, we identified specific variants from the genome-wide comparison of TNP-418 (the strain infecting Zora, an individual from the same social group) with other strains from branch 2F (Supplementary Table 9). Variants were manually checked and visualized in the partially covered genome from the strain infecting Woodstock using IGV software (Supplementary Table 10). Two variants not covered by high-throughput sequencing data were also selected for specific PCR screening. Primers were designed using the Primer3 web tool (http://bioinfo.ut.ee/primer3-0.4.0/) based on Mycobrowser sequences^68^ and are described in Extended Data Table 3. All PCR conditions were the same as in the *M*. *leprae* screening PCRs except for the primer sets and associated annealing temperatures.

### Ethical oversight

For chimpanzees, all data were collected in accordance with Best Practice Disease and Monitoring Guidelines developed by the Section on Great Apes, IUCN SSC Primate Specialist Group (IUCN SSC PSG SGA). The collection of samples was noninvasive. All proposed data collection and analyses adhered strictly to ethics guidelines of the Association for the Study of Animal Behaviour (UK). Ethical approval for targeted leprosy camera trap surveys and faecal sample collection at CNP, Guinea-Bissau, was granted by the University of Exeter, UK. The Institute for Biodiversity and Protected Areas in Guinea-Bissau approved and collaborated directly on all aspects of this research. Ethical approval for the work by the Taï Chimpanzee Project at TNP was given by the Ethics Commission of the Max Planck Society. The Centre Suisse de Recherches Scientifiques en Côte d’Ivoire collaborates on the research at TNP.

For human participants, this study was carried out under the ethical consent of the World Health Organization Global Leprosy Program surveillance network. All human participants gave written informed consent in accordance with the Declaration of Helsinki.

### Reporting summary

Further information on research design is available in the Nature Research Reporting Summary linked to this paper.

## Online content

Any methods, additional references, Nature Research reporting summaries, source data, extended data, supplementary information, acknowledgements, peer review information; details of author contributions and competing interests; and statements of data and code availability are available at 10.1038/s41586-021-03968-4.

### Supplementary information

Supplementary Information This file contains supplementary table and video titles, Supplementary Notes 1–7 and Supplementary References.

Reporting Summary

Peer Review File

Supplementary Tables 1–10

Supplementary Video 1 Videoclip shows adult female chimpanzee, Rita, with claw hand at CNP (dated April 2017).

Supplementary Video 2 Videoclip shows adult female chimpanzee, Rita, at CNP (dated May 2018).

Supplementary Video 3 Videoclip shows adult female chimpanzee, Rita, at CNP (dated November 2018).

Supplementary Video 4 Videoclip shows adult male chimpanzee, Woodstock, at TNP biting his fingernails (dated April 2019).

Supplementary Video 5 Videoclip shows adult male chimpanzee, Woodstock, with claw hand at TNP (dated July 2020).

## Figures and Tables

**Extended Data Table 1:**
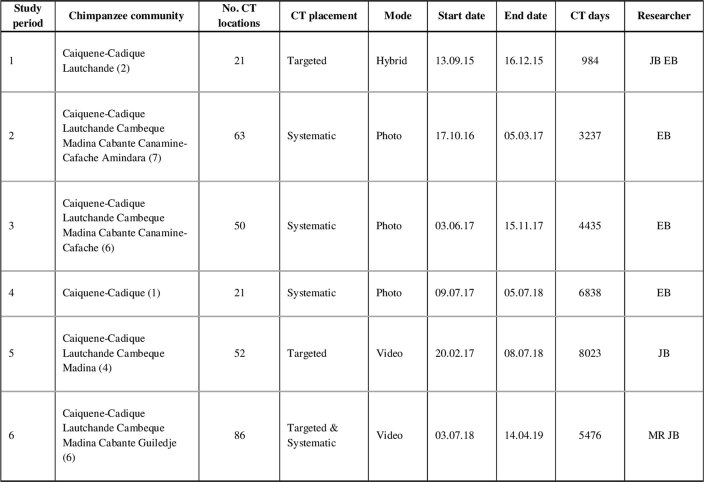
The camera trap (CT) study periods with the focal chimpanzee community within Cantanhez National Park

The number of distinct CT locations for that study period is included (total number of CT locations = 211). Certain CT locations were used in more than one study period (Supplementary Table 1). For targeted CT placement if no chimpanzees were filmed for a certain period CTs were repositioned; hence not all cameras were working at the same time. The placement design of CTs was targeted or systematic. Targeted CTs were deployed to maximize detection of chimpanzees (such as chimpanzee drumming sites, fruiting trees and trails). Systematic CTs were placed following a survey design maximizing independence between CT sites and chimpanzee detection. The CT mode was either set to photograph or video or both (hybrid) and CTs were active for 24 h per day. The start and end dates of each study period are included as well as the number of CT days. CT days are the sum of number of days for each active CT after removing days when cameras were inactive due to malfunctioning, batteries running out, trees falling in front of the CT or theft (total CT days = 28,993). The researcher initials are included (J.B., Joana Bessa; E.B., Elena Bersacola; M.R., Marina Ramon).

**Extended Data Table 2:**
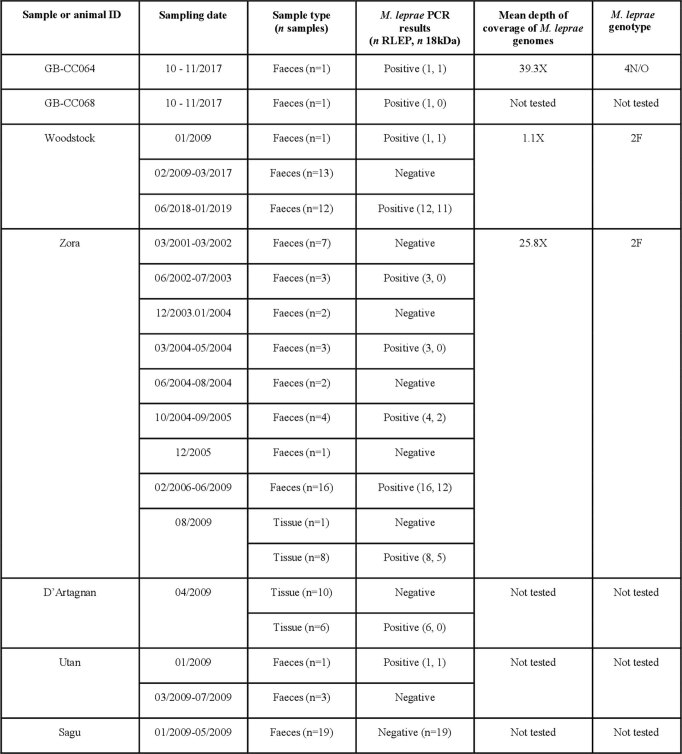
Samples (CNP) and animals (TNP) that tested positive for *M. leprae* DNA

Samples are considered as PCR-positive if they were positive either in the RLEP or in the RLEP and the 18-kDa assay (see Supplementary Table 2 for additional information).

**Extended Data Table 3:**
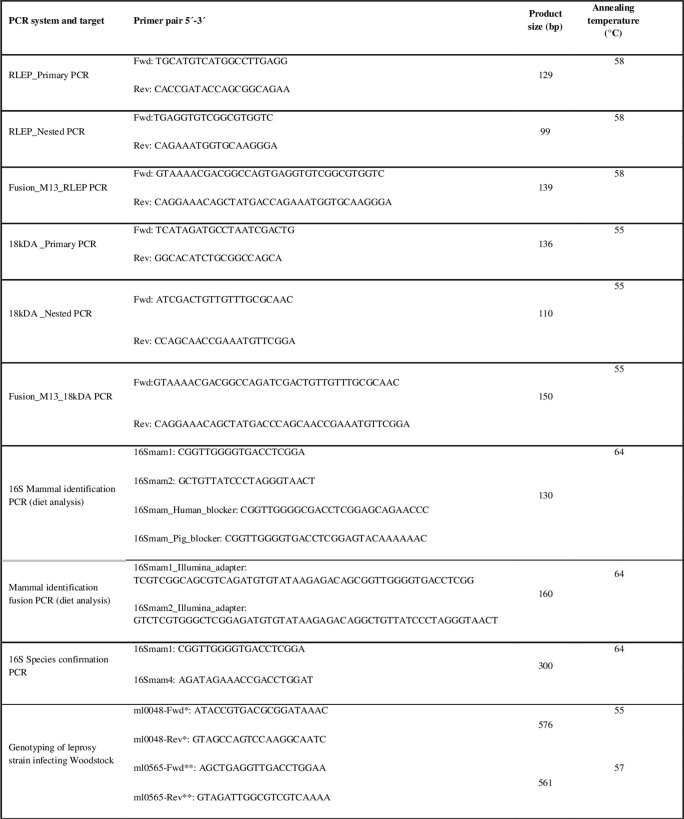
Primers used for the identification of *M. leprae* in chimpanzee tissues and faeces, diet analysis, the genotyping of *M. leprae* strains and confirmation of chimpanzee origin of the samples

Fwd, forward; Rev, reverse. *Mutation C1193T in *ml0048* (genome position 60123); a T is found in TNP-418 and TNP-566. **Mutation C319T in *ml0565* (genome position 683097); a T is found in TNP-418 and TNP-566.

## Data Availability

Sequence data are available from the National Center for Biotechnology Information Sequence Read Archive, BioProject (PRJNA664360) and BioSample (16207289–16207321). BioSample codes for all samples used in this study are given in the Supplementary Data. Other relevant data are available in the Article and its Supplementary Information.
