# Supplementary material for: Leprosy in wild chimpanzees
Source: Nature. 2021 Oct 13;598(7882):652–6. doi: 10.1038/s41586-021-03968-4 (PMC8550970; doi:10.1038/s41586-021-03968-4)
Supplement: Supplementary file 2 — Reporting Summary [file 41586_2021_3968_MOESM2_ESM.pdf]

nature research

Corresponding author(s): Fabian H Leendertz; Kimberley J Hockings

Last updated by author(s): 22/7/21

Reporting Summary

Nature Research wishes to improve the reproducibility of the work that we publish. This form provides structure for consistency and transparency in reporting. For further information on Nature Research policies, see Authors & Referees and the Editorial Policy Checklist.

Statistics

For all statistical analyses, confirm that the following items are present in the figure legend, table legend, main text, or Methods section.

n/a	Confirmed	
☐	☒	The exact sample size (*n*) for each experimental group/condition, given as a discrete number and unit of measurement
☐	☒	A statement on whether measurements were taken from distinct samples or whether the same sample was measured repeatedly
☒	☐	The statistical test(s) used AND whether they are one- or two-sided<br>*Only common tests should be described solely by name; describe more complex techniques in the Methods section.*
☐	☒	A description of all covariates tested
☐	☒	A description of any assumptions or corrections, such as tests of normality and adjustment for multiple comparisons
☐	☒	A full description of the statistical parameters including central tendency (e.g. means) or other basic estimates (e.g. regression coefficient) AND variation (e.g. standard deviation) or associated estimates of uncertainty (e.g. confidence intervals)
☒	☐	For null hypothesis testing, the test statistic (e.g. *F*, *t*, *r*) with confidence intervals, effect sizes, degrees of freedom and *P* value noted<br>*Give P values as exact values whenever suitable.*
☐	☒	For Bayesian analysis, information on the choice of priors and Markov chain Monte Carlo settings
☒	☐	For hierarchical and complex designs, identification of the appropriate level for tests and full reporting of outcomes
☒	☐	Estimates of effect sizes (e.g. Cohen's *d*, Pearson's *r*), indicating how they were calculated

Our web collection on statistics for biologists contains articles on many of the points above.

Software and code

Policy information about availability of computer code

Data collection	No software was used
Data analysis	All raw reads were adapter- and quality-trimmed with Trimmomatic v0.35. The quality settings were “SLIDINGWINDOW:5:15 MINLEN:40”. Paired-end (PE) data were additionally processed with SeqPrep (https://github.com/jstjohn/SeqPrep) to merge overlapping pairs. Preprocessed reads were mapped onto the M. leprae TN reference genome (GenBank AL450380.1) with Bowtie2 v2.2.5. SNP calling was done using VarScan v2.3.9. To avoid false-positive SNP calls the following cutoffs were applied: minimum overall coverage of five non-duplicated reads, minimum of three non-duplicated reads supporting the SNP, mapping quality score >8, base quality score >15, and a SNP frequency above 80%. InDel calling was done using Platypus v0.8.1 followed by manual curation. We used the Integrative Genomics Viewer v 2.8.13 and Basic Local Alignment Search Tool (BLAST) v 2.11.0+. Dating analyses were done using BEAST2 v2.5.2.

For manuscripts utilizing custom algorithms or software that are central to the research but not yet described in published literature, software must be made available to editors/reviewers. We strongly encourage code deposition in a community repository (e.g. GitHub). See the Nature Research guidelines for submitting code & software for further information.

Data

Policy information about availability of data

All manuscripts must include a data availability statement. This statement should provide the following information, where applicable:

- Accession codes, unique identifiers, or web links for publicly available datasets
- A list of figures that have associated raw data
- A description of any restrictions on data availability

Sequence data are available from the NCBI Sequence Read Archive (SRA) Bioproject PRJNA664360 Biosamples SAM16207289-16207321. Biosample codes for all

samples used in this study are given in the Supplementary Data. Other relevant data supporting the findings of the study are available in this published article and its Supplementary Information files.

Field-specific reporting

Please select the one below that is the best fit for your research. If you are not sure, read the appropriate sections before making your selection.

☐ Life sciences ☐ Behavioural & social sciences ☒ Ecological, evolutionary & environmental sciences

For a reference copy of the document with all sections, see nature.com/documents/nr-reporting-summary-flat.pdf

Ecological, evolutionary & environmental sciences study design

All studies must disclose on these points even when the disclosure is negative.

Study description	Study description – We report on leprosy-like lesions in two wild populations of western chimpanzees in the Cantanhez National Park (CNP), Guinea-Bissau, and the Taï National Park (TNP), Côte d'Ivoire, West Africa. We screen chimpanzee faecal and necropsy samples for the presence of M. leprae and conduct phylogenomic comparisons with other strains from humans and other animals.
Research sample	The research sample is represented by two populations of wild chimpanzees (Pan troglodytes verus) in CNP and TNP. We conducted this study in these two populations in response to leprosy-like lesions observed during behavioural monitoring. We did not discriminate between age and sex classes, instead we collected data on as many individuals as possible for analysis of leprosy symptoms. Analyses in this paper focus on symptomatic individuals. These two chimpanzee populations include male and female individuals and age estimates range from newborn to adult (~40 years of age). There are a minimum of 12 chimpanzee communities at CNP, all unhabituated to researchers, with approximately 35-60 individuals per community (age and sex composition of all communities unknown). At one community (Caiquene-Cadique), we estimate at least 48 individuals, including 16 adult females, 13 adult males, 3 subadults and 16 immatures (juveniles and infants). At TNP, the three human-habituated chimpanzee communities include a total of 91 individually recognised chimpanzees.
Sampling strategy	We performed non-invasive sampling through the collection of faeces from symptomatic and asymptomatic chimpanzees at CNP and TNP. In CNP, where chimpanzees are not habituated to human observers, this is performed by collecting faecal material found under chimpanzee nests or in proximity to chimpanzee signs (e.g. food remains or knuckle prints). At the time of faecal collection, the identity of the chimpanzee was not known. At CNP, camera traps were deployed at 211 locations including across different habitat types within the home range of eight of the 12 putative chimpanzee communities. Targeted camera traps were deployed to record and monitor chimpanzee behaviour and disease occurrence. Systematic camera traps were deployed across central CNP at a minimum distance of 1km between sampling points. At TNP chimpanzees are followed by researchers on a daily basis and faeces are collected right after observing defecation. In both cases, faeces are collected with the aid of a plastic or wooden spatula and placed in 2ml or 15ml tubes dry or with RNAlater. For this study we analysed all available faecal samples from individuals which displayed clinical signs of leprosy and optimal sample sizes could not be determined beforehand. For TNP we included only samples from the South community since leprosy was observed only in members of this chimpanzee community. Necropsies on dead chimpanzees were performed by trained veterinarians at TNP as part of the health monitoring program. For this study, we tested all available chimpanzee necropsy samples in our collection.
Data collection	Data collection was performed by local field assistants, researchers and veterinarians working at CNP and TNP. At CNP, clinical data on unhabituated chimpanzees were collected using camera traps and faecal samples were collected with the aid of a wooden spatula and stored at ambient temperature in 15ml tubes containing NAP buffer. At TNP, data were collected by research assistants both on paper sheets and using the Cybertracker app, and by veterinarians who documented via pictures and videos. At TNP, the long-term health monitoring program includes continuous collection of faecal and urine samples from known adult chimpanzees. Faeces are transferred in 2ml cryotubes with the aid of a plastic spatula and frozen in liquid nitrogen. A full necropsy is systematically performed on chimpanzees found dead by the on-site veterinarian. Tissue samples of several internal organs are taken if the state of carcass decomposition allows.
Timing and spatial scale	Camera traps were set up over six data collection periods ranging from 2015 to 2019 across CNP (1067 km2). There were six study periods in total: (1) 13.09.15-16.12.15 (984 camera trap (CT) days, targeted CT placement, 2 communities); (2) 17.10.16-05.03.17 (3237 CT days, systematic, 7 communities); (3) 03.06.17-15.11.17 (4435 CT days, systematic, 6 communities); (4) 09.07.17-05.07.18 (6838 CT days, systematic, 1 community); (5) 20.02.17-08.07.18 (8023 CT days, targeted, 4 communities); (6) 03.07.18-14.04.19 (5476 CT days, targeted and systematic, 6 communities). Data collection was stopped once we had obtained sufficient camera trap footage to determine leprosy presence across chimpanzee communities. Since 2020, the Cantanhez Chimpanzee Project has continued monitoring the health of this population. At TNP sample collection for the project started in 1994 and has been routinely carried out ever since. Over 25 years we have accumulated a collection of chimpanzee faecal and urine samples and necropsy samples from all wildlife found dead in the area. For this study, we tested samples collected between 1998 and 2019.
Data exclusions	No specifica data were excluded from the study.
Reproducibility	To confirm our results of leprosy infection we used two PCR systems in parallel and tested several samples for each individual/community. Positives were then further confirmed via next generation sequencing. For this purpose several individual libraries were generated to confirm M. leprae DNA presence in the samples.
Randomization	Randomization is not relevant for this type of study, which is based on investigating infectious causes of illness in wildlife. To maximize our chances of pathogen detection we sampled all individuals, whenever possible.

Blinding | Not applicable to this study since this is a study on a naturally occurring disease in wild animals.

Did the study involve field work? ☒ Yes ☐ No

Field work, collection and transport

Field conditions

Guinea-Bissau (36,125 km2), West Africa, lies within the Guinean forest-savannah mosaics, a biodiverse ecoregion buffering the Guinean moist forests in the south and the West Sudanian savannah in the north. The climate in Guinea-Bissau is characterized by a rainy season from mid-May to the end of October and a long dry season from November to mid-May. Cantanhez NP (N11° 14.287′ W15° 02.281′) comprises the Cubucaré peninsula in the Tombali Region bordering Guinea-Conakry. The landscape in Cantanhez NP consists of a mosaic of coastal sub-humid forest patches, mangroves, savannah grassland, woodland and agriculture including mostly cashew orchards, shifting cultivation fields and mangrove swamp rice fields. Approximately 24,000 people across 200 villages and settlements are present inside the park. The TNP (5,082 km 2), located in the south-west of Ivory Coast bordering Liberia (N5° 38 56 W7° 05 43), consists of an evergreen lowland rainforest and is the largest remaining primary forest fragment in West Africa. It is home to a wide range of mammals that include 11 different nonhuman primate species. There are no settlements or agricultural areas inside the National Park. The climate in TNP is characterized by a rainy season from March/April to the end of October and a dry season from November to February/March.

Location

Tai National Park, Ivory Coast and Cantanhez National Park, Guinea Bissau

Access and import/export

Research conducted at CNP is authorised by the Institute for Biodiversty and Protected Areas (IBAP) in Guinea-Bissau, who are partners and co-authors on this research. All research at TNP is conducted under the umbrella of a collaboration with Ivorian partners and health authorities. Samples are routinely exported to Germany for diagnostic purposes following international guidelines and prior official authorization through CITES permits, where necessary. CITES permits for importing necropsy samples from Ivory Coast are reguslary issued to the RKI. The most recent ones were issued on March 30th 2021 under the number DE-E-05895/20 and DE-E-05896/20.

Disturbance

All activities conducted for this study were carried out as part of the Cantanhez Chimpanzee Project and the Tai Chimpanzee Project. All samples and observations collected are done with the minimum disturbance to wildlife and the environment. At CNP, camera traps are used to collect data and cause minimum disturbance to chimpanzees. Faecal samples are collected when animals are no longer at the site. At TNP, a minimum distance of 7 meters is maintained from chimpanzees and samples are collected after the animals have moved away. Only non-invasive samples such as faeces and urine are collected.

Reporting for specific materials, systems and methods

We require information from authors about some types of materials, experimental systems and methods used in many studies. Here, indicate whether each material, system or method listed is relevant to your study. If you are not sure if a list item applies to your research, read the appropriate section before selecting a response.

Materials & experimental systems

n/a	Involved in the study
☒	☐ Antibodies
☒	☐ Eukaryotic cell lines
☒	☐ Palaeontology
☐	☒ Animals and other organisms
☐	☒ Human research participants
☒	☐ Clinical data

Methods

n/a	Involved in the study
☒	☐ ChIP-seq
☒	☐ Flow cytometry
☒	☐ MRI-based neuroimaging

Animals and other organisms

Policy information about studies involving animals; ARRIVE guidelines recommended for reporting animal research

Laboratory animals

This study did not involve laboratory animals.

Wild animals

At CNP, chimpanzees are not habituated to human observers and all data are collected remotely using camera traps. The age and sex distribution of chimpanzees within this population have not be calculated (as this requires accurately identifying all individuals). At one community (Caiquene-Cadique), we estimate at least 48 individuals, including 16 adult females, 13 adult males, 3 subadults and 16 immatures (juveniles and infants). At TNP, wild chimpanzee communities have been habituated by researchers since 1979. A team of field assistants and researchers follow the animals on a daily basis from a 7-meter distance, recording behavioural data and collecting faeces and urine samples whenever possible. In normal situations, each assistant or researcher has one focal individual per day to collect data and samples from. In disease outbreak situations, monitoring efforts are reinforced and sampling is attempted from all symptomatic and asymptomatic individuals. These populations include male and female individuals and estimation of age range is from newborn to adult (~40 years of age). As of March 2021, there are 91 individuals (40 males and 51 females), including 43 adults (14 males and 29 females), 5 adolescents (4 males, and 1 female), 19 juveniles (6 males and 13 females), and 24 infants (16 males and 8 females).

Field-collected samples

At CNP, chimpanzee faecal samples are collected by visiting chimpanzee nesting and feeding sites. Faecal samples were stored at room temperature in 15ml tubes containing NAP buffer, and shipped to Robert Koch Institute in Germany. At TNP, samples are

	collected upon defecation or urination of the chimpanzees and stored in 2ml cryotubes. The research camps of the Tai Chimpanzee Project are equipped with liquid nitrogen tanks for storage of samples. Samples are then transported to Abidjan for temporary storage at the Centre Suisse de Recherches Scientifiques and subsequently shipped to RKI on dry ice whenever someone is traveling. Since these samples were collected from wild living animals, no other parameter needs to be specified (e.g. housing or photoperiod).
Ethics oversight	All data were collected in accordance with Best Practise Disease and Monitoring Guidelines of the Great Ape Section of IUCN Primate Specialist Group. The collection of samples was strictly non-invasive. All proposed data collection and analyses adhered strictly to ethics guidelines of the Association for the Study of Animal Behaviour (UK). Ethical approval for targeted leprosy camera trap surveys and faecal sample collection at CNP, Guinea-Bissau, was granted by the University of Exeter, UK. The Institute for Biodiversity and Protected Areas (IBAP) in Guinea-Bissau approved and collaborated directly on all aspects of this research. Ethical approval for the work at Tai Chimpanzee Project was given by the Ethics Commission of the Max Planck Society. The Centre Suisse de Recherches Scientifiques en Côte d'Ivoire collaborates on the research at TNP.

Note that full information on the approval of the study protocol must also be provided in the manuscript.

Human research participants

Policy information about studies involving human research participants

Population characteristics	M. leprae strains were collected from skin samples of newly diagnosed patients with positive bacillary index. These were obtained from the respective National Leprosy Control Programs in the framework of the leprosy drug resistance surveillance programs. Among the 21 patients included retrospectively in this study, seven were female and 13 were male (one unknown), ranging from 18 to 80 years in age. They originated from Mali (n=8), Benin (n=6), Niger (n=5), Côte d'Ivoire (n=1) and Senegal (n=1).
Recruitment	Patients were not recruited for this study. Inform consent were collected by the respective National Leprosy Control Programs during diagnosis to allow the use of the M. leprae strain genetic informations.
Ethics oversight	This study was carried out under the ethical consent of the WHO Global Leprosy Program surveillance network. All subjects gave written informed consent in accordance with the Declaration of Helsinki.

Note that full information on the approval of the study protocol must also be provided in the manuscript.
